# Can mobile-bearing unicompartmental knee arthroplasty achieve natural gap-balancing? An observational study with a novel pressure sensor

**DOI:** 10.1186/s13018-022-03255-6

**Published:** 2022-09-05

**Authors:** Shaokui Nan, Zheng Cao, Yue Song, Xiangpeng Kong, Haifeng Li, Wei Chai

**Affiliations:** 1 Senior Department of Orthopedics, The Fourth Medical Center of PLA General Hospital, No. 51 Fucheng Road, Beijing, 100048 China; 2 National Clinical Research Center for Orthopedics, Sports Medical and Rehabilitation, Beijing, China

**Keywords:** Mobile bearing, Unicompartmental knee arthroplasty, Pressure sensor, Soft tissue balancing, Clinical outcomes

## Abstract

**Background:**

Mobile-bearing unicompartmental knee arthroplasty (MB-UKA) is an effective treatment for anteromedial knee osteoarthritis. Meticulous intraoperative soft tissue balancing remains challenging yet consequential for a successful operation. Currently, surgeons rely mostly on their experience during soft tissue balancing, yielding unreproducible results. The purpose of this study was to quantified measure the soft tissue tension of medial compartment and determine if an optimal "target" tension values with the natural state exists.

**Methods:**

This was an observational study of 24 consecutive patients. All 30 UKAs were performed by a single surgeon. The piezoresistive sensor was custom designed to fit in the medial compartment gap. Contact pressures were measured at 5 angular positions of the knee intraoperatively: 0°, 20°, 45°, 90°, and 110° of flexion. The change in pressure from extension (20° position) and flexion (110° position) was also calculated (E-FPD). Data on age, sex, body mass index, operative side, and bearing size were collected. Outcome measures were measured at baseline and at the 6-month postoperative follow-up; Oxford Knee Score, visual analog scale score, and range of motion were compared to evaluate clinical outcomes.

**Results:**

There was a significant improvement in patients in all measured outcomes at 6 months from baseline (*P* < 0.05). The E-FPD of 14.9 N (8.9, 24.6) was indicative of appropriate soft tissue balancing throughout the functional range of knee motion. Of 30 knees, 22 were 3-mm bearing and 8 were 4- or 5-mm bearing. The pressure data of the 3-mm bearing group was larger than that of the non-3-mm bearing group for each knee flexion degree, but the difference was not statistically significant (*P* > 0.05).

**Conclusions:**

Objective data from sensor output may assist surgeons in decreasing loading variability during MB-UKA. The data suggested that MB-UKA could not accurately restore soft tissue tension to the natural state, which was related to the inability of MB-UKA surgical instruments to fine adjust the bone cut and soft tissue release.

***Study registration*:**

Chinese Clinical Trial Registry (http://www.chictr.org.cn): ChiCTR1900024146.

**Supplementary Information:**

The online version contains supplementary material available at 10.1186/s13018-022-03255-6.

## Background

Unicompartmental knee arthroplasty (UKA) is considered an alternative therapy for anteromedial knee osteoarthritis and osteonecrosis if conservative treatment is noneffective [1–3]. Consequently, UKA yields a better range of motion and joint function as seen in early follow-ups than TKA [4–8]. Therefore, Mobile-bearing UKA (MB-UKA) represented by the Oxford system has slowly gained global popularity.

Based on standard surgical technology and optimized surgical instruments, MB-UKA provides joint surgeons precise bone resection under the premise of loose tolerance for prosthesis orientation [9, 10]. Surgeons aim to achieve recovery of the medial soft tissue’s natural tension. However, till date, there is no concept to define natural tension in the medial compartment, and surgeons’ experience determined by the soft tissue balance, which varies considerably among individuals. This poses a challenge in performing MB-UKA, as unsuitable tension could affect early clinical outcomes.

Pressure sensors have been used to provide a quantitative measure of soft tissue tension in TKAs, assisting surgeons in achieving an appropriate balancing of the flexion and extension gap and satisfactory clinical outcomes [11–15]. Regarding UKA, Sun et al. [16] reported the use of a pressure sensor beneath the UKA trial bearing on processed fresh frozen cadaveric specimens; Mentink et al. [17] embedded a sensor within the bearing of MB-UKA and determined its electrical and mechanical characteristics by in-vitro test. To our knowledge, clinical research of pressure sensors to measure soft tissue tension during MB-UKA procedures has not previously been reported. We designed a type of pressure sensor for MB-UKA to test soft tissue tension in the medial compartment. We assumed that experienced surgeons could accomplish natural tension balance manually. The purpose of this study was to quantified measure the soft tissue tension of medial compartment which was balanced by an experienced surgeon and determine if an optimal "target" tension values with the natural state exists (Additional file 1).

## Methods

### Design, setting and participants

This was a prospective, observational study conducted in a single orthopedic center. Patients who underwent unilateral or bilateral MB-UKA between January 2021 and June 2021 were considered eligible. The inclusion criteria were a radiographic diagnosis of isolated medial compartmental osteoarthritis or idiopathic osteonecrosis with fixed flexion deformity of < 15°, an active ROM over 90°, and a varus deformity of < 15°. The exclusion criteria were inflammatory arthropathy, knee lateral compartment degeneration, anterior cruciate ligament dysfunction, and Lateral subluxation of the patellofemoral joint. In total, 24 patients (30 UKAs) met our inclusion criteria and were prospectively enrolled in the trial. Demographic characteristics, including age, sex, body mass index, diagnosis, and operative side, were collected. All procedures were performed by one senior joint surgeon who has completed about 300 cases of UKA (about 60 cases per year) using the third-generation Oxford UKA prosthesis (Zimmer, Biomet, Warsaw, IN, USA) for all knees.

### Ethics, consent, and permissions

The methods and procedures for our study were approved by our institutional Ethics Review Committee. All patients were informed of the use of intraoperative sensors to quantify soft tissue balancing during the MB-UKA procedure. All participants provided signed informed consent for the procedure and for the use of their data for research purposes. All procedures were performed in accordance with the World Medical Association Declaration of Helsinki.

### Description of the pressure sensor

**Fig. 1:**
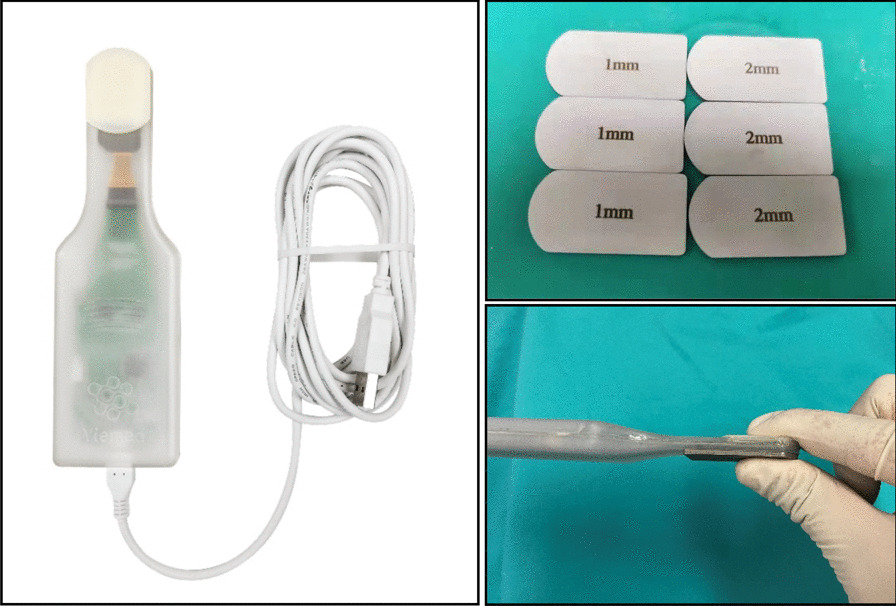
Design of the custom-designed contract pressure sensor, including the metal sheet for the tibial side

The pressure sensor consisted of an electronic measuring gasket (Yiemed Co. Ltd., Shandong, China), with a data cable connecting to a computer for data acquisition (Fig. 1). The bearing, which is the core component of the sensor, was fabricated in the shape of the mobile-bearing spacer for the Oxford UKA prosthesis. Intraoperatively, the bearing was inserted into the medial gap and contact pressure was measured at 5 fixed angular positions of the knee joint (0°, 20°, 45°, 90°, and 110° of flexion). Notably, the average width of the medial gap for an Oxford UKA prosthesis is 3 mm. As the sensor had a width of 6 mm, the tibial trial component of the prosthesis (generally 3 mm in height) was removed for appropriate positioning of the sensor. To ensure an appropriate fit, 1-mm metal sheets were used on the tibial side. Analog pressure values (mV) were converted to digital values (N), using an analog-to-digital converter chip in the sensor, and transferred to the computer for analysis.

### MB-UKA surgical procedure

**Fig. 2:**
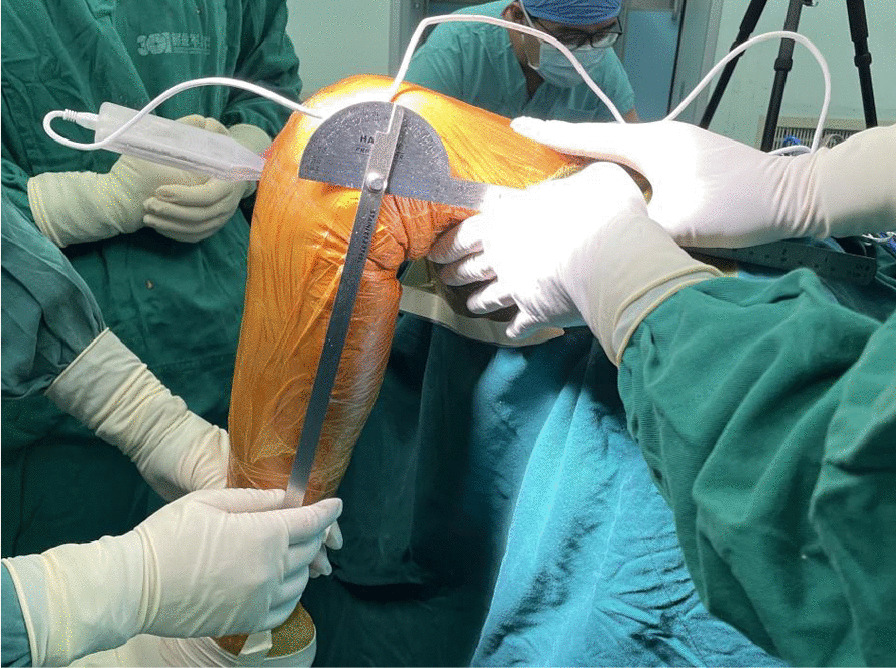
Representative case showing the intraoperative use of the contact pressure sensor at predetermined angular positions of the knee

All procedures were performed under general anesthesia, with the patient placed in the standard half-lithotomy position as described in the manufacturer’s guidelines. A parapatellar medial approach was used. The anterior cruciate ligament and lateral compartment cartilages were examined intraoperatively. Based on our relatively strict indications, there was no indication to shift from MB-UKA to TKA intraoperatively for any patient. After removal of any osteophyte along the joint line and bone resection, the trial tibial and femoral implants were inserted, and the soft tissue was balanced based on the surgeon’s expert judgment using the “two-finger” method at two knee joint positions, namely 20° and 110° of knee flexion. The tibial trial implant was then removed and the pressure sensor inserted in the medial gap, with 1-mm metal sheets used on the tibial side to achieve a good fit. The contact pressure was recorded at 5 specific angular positions of knee joint flexion, 0°, 20°, 45°, 90°, and 110°, with three insertions to obtain three pressure recordings at each position. The knee flexion angle was determined by long-arm goniometer. The contact pressure difference over an arc of extension to flexion of 90° (E-FPD) was quantified as the difference in contact pressure between the 20° flexion and the 110° positions. For all measurements, the lower limb was maintained in a neutral position (no varus or valgus), with the movement guided by support provided on the posterior surface of the ankle to avoid external compressive forces (Fig. 2). Notably, no intraoperative decisions to revise the bone cuts or change the implant size were made based on results of contact pressure analysis.

The 10-point pain visual analog scale (VAS), Oxford knee score (OKS), and range of motion (ROM) were collected preoperatively (baseline) and at the 6-month follow-up appointment. A validated version of the Chinese version of the OKS was used to evaluate postoperative knee function. Improvement in knee ROM was used as an assessment of clinical outcomes. The change in these values at 6 months from baseline were used in the analysis.

### Sample size

Sample size of the trial was based on the ability to detect minimal clinically important difference (MCID) of OKS in knee replacement, which was reported as 4.7 to 10 points in Maredupaka et al.’s study [18]. With the *α* level of 0.05 and test power of 80%, at least 5–11 patients were needed in our trial.

### Statistical analysis

All statistical analyses were performed using SPSS version 22 software (IBM Corp., Armonk, NY, USA). Normality of data distribution was assessed using the Kolmogorov–Smirnov test. Data following normal distribution are presented as mean ± SD and medians and interquartile ranges (IQRs) if non-normally distributed. Reliability was calculated using the intraclass correlation coefficient (ICC). ICC estimates and their 95% confidence interval (95% CI) were calculated with a two-way mixed-effects model for absolute agreement. A paired sample t-test was used to evaluate the significance in the difference scores at 6 months from baseline. We divided the patients into two groups, 3 mm bearing group and non-3 mm bearing group. We performed subgroup analysis to compare the data of both groups.

## Results

### Participants

The study group included 24 patients, 5 men, with an average age of 64.9 ± 7.7 years and average body mass index of 24 ± 6.3 kg/m^2^. The full demographics of our study group are reported in Table 1.

**Table 1:** Demographic characteristics of the study group at baseline

Age (years)		64.9 ± 7.7)	
Sex (Male/female)		(5/19)	
BMI (kg/m^2^)		24 ± 6.3	
Operation side (left/right)		13 knees/17 knees	
Bearing size	3 mm	4 mm	5 mm
	22 knees	5 knees	3 knees

### Intraoperative sensor data

**Fig. 3:**
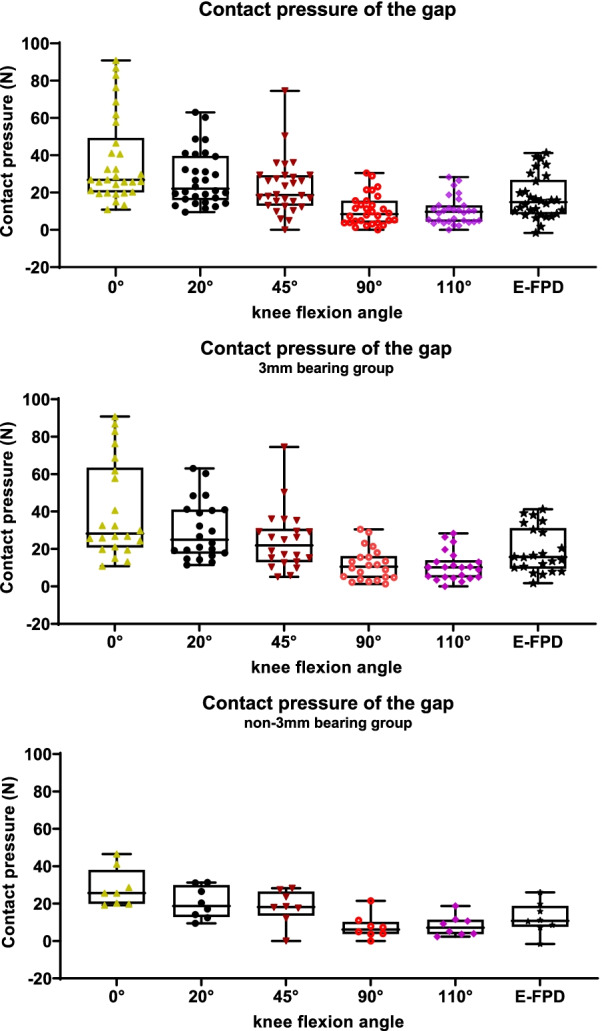
Box plots of the distribution of contact pressure measures at the predetermined angular positions of the knee

The contact pressure measures at each of the knee joint angular positions are reported in Table 2 and Fig. 3. These data do not have a normal distribution (alpha = 0.05). The ICC values between the three measurements shows good reliability (ICC > 0.9). The pressure data of the 3 mm bearing group was larger than that of the non-3 mm bearing group for each knee flexion degree, and the data dispersion of the 3 mm group was greater, but the Kruskal–Wallis test analysis of the two groups of data showed no statistically significant difference.

**Table 2:** Contact pressure measures at each of the knee joint angular positions

Flexion	Pressure (N)			
	Total (n = 30)	3-mm bearing group (n = 22)	Non-3-mm bearing group (n = 8)	*P* value
0°	26.8 (20.5,45.2)	28.3 (21.9, 60.7)	25.6 (20.1, 31.7)	0.360
20°	22.1 (16.6, 37.5)	22.1 (18.2, 40.9)	18.7 (13.7, 27.7)	0.113
45°	18.7 (13.6, 29.0)	21.9 (13.6, 29.3)	18.2 (16.2, 24.5)	0.542
90°	8.4 (4.1, 15.0)	10.5 (4.8, 15.6)	6.1 (3.8, 8.7)	0.223
110°	9.7 (4.6, 12.6)	10.2 (5.0, 13.1)	7.2 (3.8, 10.9)	0.425
E-FPD	14.9 (8.9, 24.6)	14.8 (8.5, 33.0)	10.8 (8.2, 16.8)	0.291

*P* value: 3-mm bearing group compared with the non-3-mm bearing group

### Outcome measures

Measured outcomes at the 6-month follow-up are summarized in Table 3. The change in the OKS and VAS scores from baseline to 6 months postoperatively was significant (*P* < 0.05). No intraoperative complications or postoperative adverse events were reported.

**Table 3:** Outcomes measures at baseline (preoperatively) and at the 6-month postoperative follow-up

	Pre-op	Post-op	*P* value
Extension (degrees)	4.7 ± 4.1	4.0 ± 3.0	0.344
Flexion (degrees)	118.8 ± 10.5	124.1 ± 10.2	0.024*
ROM (degrees)	114.1 ± 11.5	120.0 ± 10.3	0.016*
VAS	6.2 ± 1.8	1.2 ± 0.9	0.000**
OKS	25.5 ± 8.6	39.7 ± 4.4	0.000**

*P < 0.05; **P < 0.01

## Discussion

We designed, manufactured, and multiply evaluated a novel sensor device that can digitally monitor and record the tibiofemoral forces during MB-UKA. Our findings demonstrate the usefulness of our sensor to provide quantitative measures of soft tissue balancing, from extension to flexion, intraoperatively, with satisfactory clinical outcomes obtained at the 6-month follow-up and no intra- or postoperative complications noted. In addition, we found that the pressure data of the 3 mm bearing group was larger than that of the non-3 mm bearing group for each knee flexion degree and the data dispersion of the 3 mm group was greater.

For MB-UKA, appropriate soft tissue balancing from knee flexion to extension is necessary to lower the risk of complications. In practice, the “two-finger” method is generally used to judge the tension of soft tissue through the range from knee extension to flexion. However, this method lacks accuracy, being largely influenced by the surgeon’s experience. As such, an objective intraoperative measure of soft tissue tension is desirable as the resultant tension directly influences postoperative outcomes of UKA.

The usefulness of sensors for soft tissue balancing has previously been reported. Surgeons have observed significant improvements in soft tissue balancing with the use of sensor-assisted knee replacement [19]. The correlation between a soft tissue balance, achieved with the use of sensor technology, and postoperative outcomes is inconsistent; while Golladay et al. [20] reported higher patient satisfaction with soft tissue balancing, Macdessi et al. [21] did not identify better short-term outcomes. Sun et al. [16] previously reported on a pressure sensor that was inserted under the bearing of the trial component which increased the actual thickness of the bearing, so their design produced relatively large fluctuations in pressure measurements within a certain range. We designed our sensor to be directly inserted into the medial compartment of the knee, which can simulate real thickness of the bearing, and the data of soft tissue tension is more approximate to the realistic situation. Studies show that the most appropriate range of soft tissue tension of TKA is 22 N–200 N[22]; however, the tension data of MB-UKA we measured was significantly less than this range. This is consistent with our clinical experience.

**Fig. 4:**
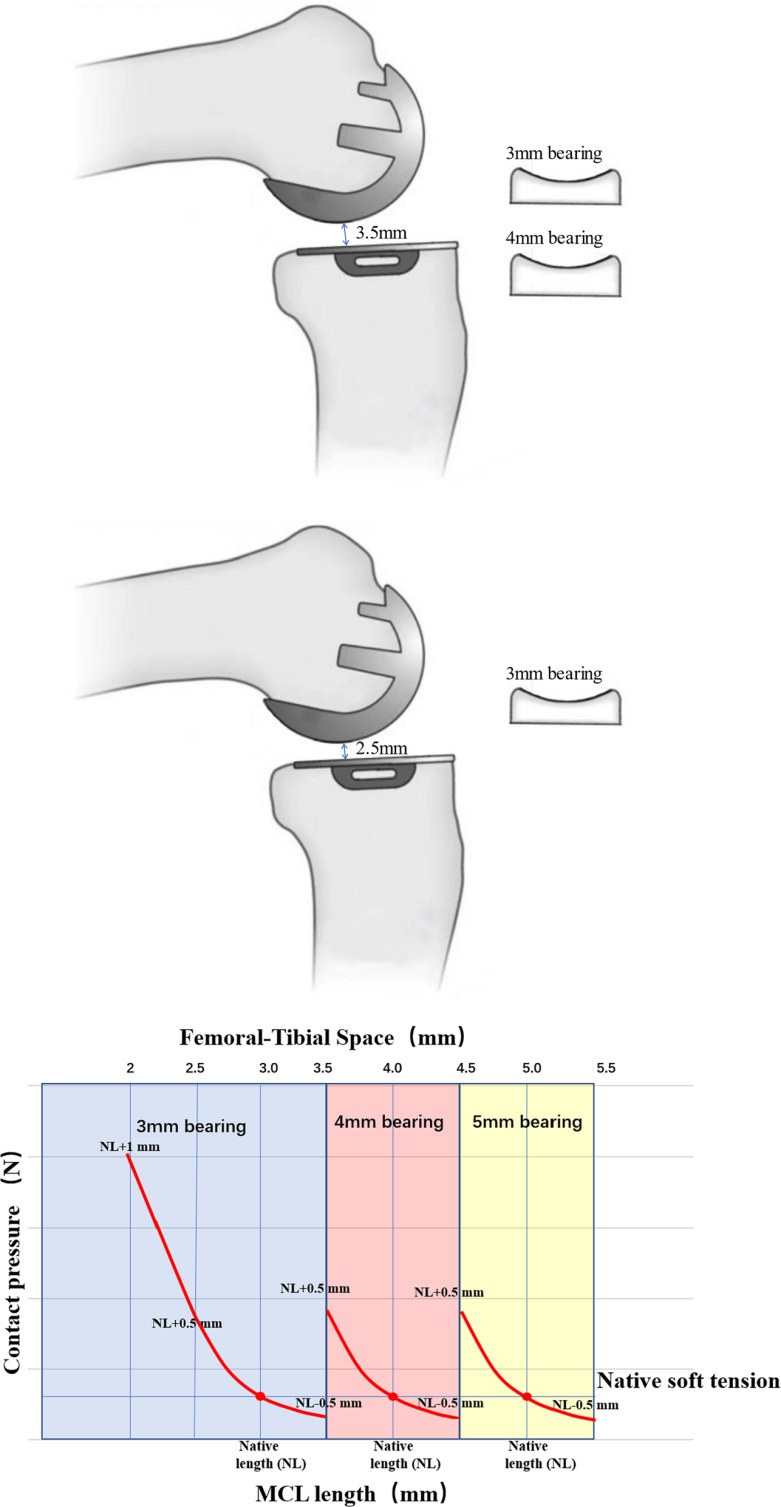
When the soft tissue tension is natively, 3 mm or 4 mm bearing can be selected if the gap of tibial-femoral component is between 3 and 4 mm, and the final tension will be slightly greater than or less than the native tension. However, when the gap is less than 3 mm, since the thinnest spacer is 3 mm, the final tension will be greater than the native tension, and there may be a significant increase (when the gap is ≤ 2 mm)

Did we accurately restore the natural soft tissue tension of the knee during MB-UKA? In the subgroup analysis, we found that the median pressure in the 3 mm group was larger than that in the non-3 mm bearing group, though the difference in the mean values was not statistically significant. Thus, clearly larger pressures were observed in the 3 mm group. This difference may be related to the use of operating instruments specifically-designed for MB-UKA. These instruments allow the surgeon to perform an osteotomy with soft tissue tension at the "natural tension." The resulting tibiofemoral gap should theoretically be 3 mm or 4 mm (for No. 3 G-clamp and No. 4 G-clamp, respectively). However, due to operational errors, the gap is often smaller or larger (Fig. 4a, b).

The lengths of the stretched ligament are not linearly correlated to the corresponding tensions, and their stress–strain curve is concave and upward [23]. During MB-UKA, if the gap is less than the target, the length of the ligament with bearing will be longer, and the contact pressures will be larger and more dispersed. When the post-resection gap is greater than the target gap, the length of the ligament with bearing is shorter, and the pressure is smaller and more concentrated (Fig. 4c). A 3 mm bearing can be selected if the gap of tibiofemoral component is between 3 and 3.5 mm, and the final tension will be slightly greater than native tension. However, when the gap is less than 3 mm, since the thinnest bearing is 3 mm, the final tension will be greater than the native tension, and there may be a significant increase (when the gap is ≤ 2 mm).

Since MB-UKA absolutely prohibits soft tissue release, adjustment of soft tissue tension can only rely on adjusting the bone cut. It is difficult to fine-tune soft tissue tension after the initial osteotomy. Because in the initial osteotomy, it is difficult to accurately control the tibiofemoral component gap exactly 3 mm, MB-UKA cannot accurately restore the soft tissue tension to the natural state and can only approach the natural state.

Therefore, in order not to make the final tension too large during MB-UKA, the target clearance of osteotomy should be set at 4 mm, and a no. 4 G-clamp should be used to obtain the final tension more easily close to the natural tension, or a new type of 3.5 G-clamp should be designed to help better obtain the soft tissue tension close to the natural tension. If in the future sensor technology can be used in combination with robot-assisted technology, the soft tissue tension could be adjusted more accurately to achieve postoperative tension after MB-UKA that is closer to that of the natural state.

The limitations of our study need to be acknowledged. First, this device is novel and our experience of using it was little, this will cause measurement errors. We will continue to test to improve it. Second, our sample size was relatively small, with a relatively large dispersion of pressure measurements as shown in Fig. 3 at each angular position. A larger sample size will be needed to provide more accurate estimates of target pressure values. Third, the surgeon’s experience with UKA was used as the reference for appropriate soft tissue balancing, which may have introduced bias. And our sensor is relatively large, the weight might be an issue. In addition, the data were transmitted via a connection cable. These features might hinder the surgical procedure and, thus, will benefit from redesign once hardware limitations are addressed. Finally, our findings will need validation using a randomized control trial design.

In summary, we used a novel sensor to obtain quantitative measurement of intraoperative medial compartment soft tissue tension, which was the natural state defined by the surgeon during the MB-UKA procedure. We found that the soft tissue tension after MB-UKA was lower than that after TKA and fluctuated across a larger range, suggesting that MB-UKA could not accurately restore the soft tissue tension to the natural state, which was related to the inability of MB-UKA surgical instruments to finely-adjusted bone cut and soft tissue release.

## Supplementary Information

**Additional file 1:** Strobe checklist.

## Data Availability

The datasets used are available from the corresponding author on reasonable request.

## Abbreviations

ICC: Intraclass correlation coefficient
IQR: Interquartile range
MCID: Minimal clinically important difference
OKS: Oxford knee score
ROM: Range of motion
TKA: Total knee arthroplasty
UKA: Unicompartmental knee arthroplasty
VAS: Visual analog scale
